# Pro-environmental behaviour is undermined by disgust sensitivity: The case of excessive laundering

**DOI:** 10.1371/journal.pone.0302625

**Published:** 2024-06-13

**Authors:** Erik Klint, Gregory Peters, Lars-Olof Johansson

**Affiliations:** 1 Department of Technology Management and Economics, Division of Environmental Systems Analysis, Chalmers University of Technology, Gothenburg, Sweden; 2 Department of Psychology, University of Gothenburg, Gothenburg, Sweden; SKUMS: Shahrekord University of Medical Science, ISLAMIC REPUBLIC OF IRAN

## Abstract

The amount of laundry washed by European consumers has grown excessively for reasons that cannot be explained by demographics alone. Initiatives trying to curb this trend have repeatedly failed. Previous studies have largely overlooked the psychological dimensions of laundering behaviour. In three separate studies we investigate how disgust, shame, cleanliness norms and environmental identity, mediated through a set of preceding behaviours, affect washing frequency. Our results highlight how conflicting psychological goals between disgust sensitivity and pro-environmental identity can undermine willingness to change laundry behaviour. Policy recommendations are suggested, and future research challenges are discussed.

## Introduction

Emissions from textile life cycles have increased considerably in the last 20 years. Recent estimates suggest that clothing consumption is responsible for 2.4–7.6% of global greenhouse emissions [1]. In addition, 16–35% of the total global emissions of microplastics comes from the laundering of synthetic fibre [2,3]. Depending on a citizen’s nationality, most of the environmental impacts of their garment life cycles can be attributed to the production or use phases. Globally, cleaning practices such as laundering are major contributors [4]. Looking at laundering in more detail, the practices of European households are fairly stable and has been estimated to 4.7 cycles per week in the year 2000 [5] and 4 cycles per week in year 2015 [6]. It is however, of concern that the average load capacity of washing machines in the EU grew considerably during the same period. Machines with a rated capacity larger than 6 kg constituted 64% of all sales in 2015, compared to 2% in 2004 [7]. Consumers consistently state that they use the full capacity of the machine [5,6,8] and the amount of laundry washed has been growing excessively beyond what can be explained by demographics [9].

Steady improvements of the technical systems associated with laundry (e.g. sources of electricity production and machine efficiency) have over time reduced environmental impacts. However, the most important determinants of final emissions from domestic laundering are still how, and how often, we choose to clean our clothes [10]. Due to this, information campaigns, interventions, and policies targeting consumer behaviour have been growing in popularity. Examples include campaigns aiming to reduce the choice of temperature and increase the use of ECO-programs. Yet, few of these initiatives seem to have had any effect in real life [11,12].

While an extensive amount of research concerns domestic laundering from a sociological or technical point of view, psychological dimensions have largely been overlooked [13]. From a technical point of view, laundering could be defined by the resources and techniques used when removing contamination from clothes. However, from a psychological perspective laundering is more tied to the notions of cleanliness and societal acceptance. While policymakers typically strive to minimize emissions by optimizing technical systems, they may unintentionally make consumers weigh perceived social risks against environmental considerations. Furthermore, people are usually reluctant to change their laundering routines [14], holding the (false) belief that laundering does not cause any environmental impacts [15], and harbouring prejudices about the effectiveness of washing at colder temperatures [12].

## Aim of the study

There is a lack of knowledge about the psychology underlying excessive laundering. This article describes an initial exploration of the psychological factors affecting household laundering decisions. Since laundering psychology is a rather niche area of concern, no relevant constructs have previously been customized and validated for this specific context, nor have any tailored psychometric tools been developed. The aim of this research is therefore to lay some of the empirical groundwork for such future attempts. Based on our results, we also aim to facilitate a more nuanced discussion about pro-environmental initiatives. We want to highlight the importance of addressing the competing psychological goals that undermine the willingness to change. Three independent studies were conducted with the overarching research question: *Can psychological aspects of cleanliness be used to explain excessive domestic laundering frequency*?

### Exploratory factors

Based on previous research [13] the following five different lines of inquiry were deemed especially interesting to pursue:

#### Disgust

Disgust is a culturally independent and universally shared emotion among humans [16]. Its main function is to protect us from diseases [17,18], although recent findings suggest more general functions such as protecting the self from offensive objects and social groups [19]. As for the potential relevancy for laundering, consider for example the qualitative field work by Curtis and Biran [20]. Here the authors explored the motivations for hygiene behaviours in different countries and found that common sources of disgust included: worn clothes (India), dirty clothes (Burkina Faso, West Africa), dust and sweat (Netherlands), and a “sweaty person” (United Kingdom). In addition to this, Reicher et al. [21] showed that in-group relations attenuate core disgust in relation to sweaty clothing. Taken together, this suggests that individuals experiencing high level of disgust towards dirty or smelly clothes may wash more often than those not experiencing such emotions.

#### Shame

Shame is often regarded as a self-conscious emotion [22,23]. This means that the emotion of shame largely contains social properties and is experienced when the individual sees themselves through the eyes of others; understanding that others judge, evaluate, and form opinions about their person [24]. As for cleanliness violations that could elicit feelings of shame, olfactory cues such as body odours [25] including signs of sweat [26,27] seem especially prominent to investigate. In other words, individuals who experience high levels of shame connected to dirty or smelly clothes might be assumed to wash more than those not experiencing such emotions. Having said that, it should be noted that there seems to be some initial evidence that the evolution of shame piggybacked on the disease avoidance architecture associated with disgust sensitivity [28]. Should this turn out to be true it would imply that including both constructs would result in an over-specified model. However, the extent of this relationship, and whether this dynamic can be observed in larger populations (or between different cultures for that matter), remains to be investigated.

#### Cleanliness norms

According to Shove [29], trends in cleanliness are a result of societal normalization of both what it means to be clean and how laundering should be done. Accordingly, configurations of domestic laundering practices could be seen as extrapolations of cultural norms combined with notions of socio-economic status of the household [26,30,31]. Previous research suggests that normative messages have the power to steer decisions and behaviour far more effectively than simply informing about any preferable action [32,33]. Prevailing psychological models measuring the influence of personal and social norms, such as the Norm Activation Model [34], have been shown to predict pro-social intentions as well as environmental behaviours [35]. Stronger individual identification with stricter norms regarding cleanliness would thus suggest a higher wash frequency.

#### Environmental identity and beliefs

Explicit pro-environmental identity can be associated with higher levels of self-reported pro-environmental behaviours and policy preferences [36]. Nevertheless, it is very hard to influence pro-environmental behaviours by simply trying to persuade people to do the right thing, e.g. consume or pollute less [32]. One reason for these types of interventions rests on the assumption that many consumers want to minimize environmental impacts from their own actions. For laundering this would suggest a common adjustment towards washing less frequently and choosing colder temperatures [4]. However, few people take sustainability into account when washing clothes [37]. Instead, low costs or good washing results are considered more important than minimizing environmental impact. The general relevance of environmental aspects concerning laundering behavioural is thus unclear. For the current investigation it is assumed that a strong environmental identity, or believing that laundering cause emissions, leads to a lower wash frequency.

#### Habits

One of the few articles that has analysed domestic laundering from a psychological perspective is Labrecque et al. [38]. Their conclusion was that adaptation of new products and subsequent behavioural change must consider pre-existing habits for successful implementation. Similar conclusions were highlighted by Conrady et al. [37] including additional barriers for change: anxieties about damaging the washing machine, anxieties connected to poor wash results, and resistance to accumulating too much dirty laundry before washing. A need to shift focus towards the believed direct consequences of laundering behaviours has also been suggested by McQueen et al. [39] as well as by Harris et al. [40], and seems like a promising avenue to investigate. In this regard the role of habits should be considered twofold. First, habits could be seen as forces driving washing (e.g. only using clothes a few times before washing them or washing on a specific day/after a specific activity regardless of the amount of dirty clothes available). Secondly, habits could also be seen as indicators of behavioural stability (e.g. consumers do not want to deviate from previous, successful, laundering decisions). As a first line of inquiry, it is hypothesized that people who exhibit a stable wash pattern wash more frequently, and that people that wash relatively empty machines wash more frequently as well.

### Potential challenges

Studies investigating the psychology underlying environmental behaviour typically rely on introspective measures that exhibit low discriminant and/or convergent validity between theoretical concepts [41,42]. Our studies are no different; all our investigated psychological factors rely on such measures. Furthermore, the construct of shame is often measured in two separate ways; shame-proneness (i.e. trait shame) and momentarily feelings of shame (i.e. state shame) given a specific situation [43]. Since we try to capture ‘state shame’ in relationship to cleanliness violations it means that the trait-aspects of the construct are unaccounted for. It also suggests that the discriminatory properties of the final construct might be even lower than initially assumed, since ‘states’ are inherently hard to capture properly [44].

In addition, a vast number of methodological challenges exist for behavioural research [45]. A remedy for some of these problems is instead to construct models measuring latent variables using both introspective and behaviour-based items [46,47]. The benefit of such an approach is that individual attitudes can be inferred from actions less prone to bias and misattribution. Yet even though attitudes [48] and values [42] are important for estimating pro-environmental behaviours, these constructs are often weak predictors of variance compared to other background variables. Income is, for example, often positively correlated with education and environmentally significant behaviours [49]. Higher income is also a prerequisite for certain types of polluting behaviours such as flying. This suggests that our psychological constructs relying on introspective measurements will exhibit relatively low predicting power. On the other side of the spectrum we expect that the background variables, such as the number of children in a household, would exhibit the strongest predicting power. Since the measurement of the habitual factors relies on manifested actions, their predicting power is assumed to be intermediate.

## Study 1

### Participants and methods

1116 individuals were recruited from a Swedish national panel to take part in an online survey, in collaboration with NOVUS (a Swedish professional analysis and research company). The data collection started at the 17^th^ of October 2022 and was completed the 24^th^ of October 2022. The invited participants were part of the NOVUS-recruited national panel, where informed consent is mandatory for participation. A written ethical approval for the work was sought from the Central Ethical Review Board in Gothenburg, who determined that the research was exempt from the requirements stated in the Swedish Ethical Review Act. The number of respondents, as well as criteria for background data, was selected so that the results would be representative for the general population in Sweden. All participants were screened based on their self-reported wash responsibility in their respective household. Individuals who claimed no responsibility at all (n = 78) or were unsure of their responsibility (n = 0), were excluded from participating in the survey. Of the remaining 1038 responses some had to be filtered out since they did not fit the limitations of the study. These observations included individuals who: primarily used a professional cleaning service for their laundry (n = 12), washed everything by hand (n = 27), or had someone else (e.g. parents, a maid, or the domestic service) wash for them (n = 7). Observations were also excluded if the respondents had failed to record their assessed number of wash programs each month (n = 56). In total, 994 observations could be used in the analysis.

In addition to basic self-reported laundering behaviours (e.g. number of washes each month, machine filling level when washing, choice of temperature, and choice of drying method), seven indexes were created which were hypothesised to have causal relationship with laundering frequency:

- **Disgust:** The inherently disgusting properties of many stains and odours motivate people to wash more frequently.
- **Shame:** The fear of a potential social stigma for wearing unclean clothes among friends and colleagues motivate people to wash more frequently.
- **Cleanliness norms:** The (assumed) interest in avoiding deviations from the current prevalent cleanliness norms (i.e. use clean clothes when you are in public spaces) motivates people to wash more frequently.
- **Environmental belief:** People who believe that laundering causes environmental emissions tend to wash less frequently (based on the assumption that they want to minimize environmental impacts).
- **Mean number of wears (habit 1):** Using clothes fewer times before washing them leads to higher washing frequencies. As for the type of clothes to be measured, the choice was made to focus on pants since this type of apparel is the most common between the genders.
- **Often wash few items (habit 2):** Running wash programs with few items (regardless of the underlying reason) will lead to an increase in wash frequency.
- **Robustness of behaviour (habit 3):** The unwillingness to change when the washing is done (e.g. every Sunday) might lead to lower filling levels and higher washing frequencies.

The complete list of specific questions used can be found in the supporting information. To minimize the risk of any misinterpretation of the questions, an initial version of the survey was presented and tested as a pilot study using a smaller convenience sample (i.e. a non-randomly selected group) which was not part of the national panel. The results from the pilot study are not included in this analysis. The data from the main survey was analysed using R (version 4.2.2).

### Results and discussion

Each index was calculated as an arithmetic mean of the respective items. The reliability (Cronbach’s alpha, α) was deemed sufficient for all indexes except for habits regarding behavioural robustness (α = 0.47) which was excluded from the continuing analysis. Since the index for ‘Wash only few items’ only consisted of a single item it was not relevant for consistency validation, see Table 1.

**Table 1:** Descriptive summaries and bivariate correlation matrix for each index.

Index category	n	Mean	SD	α	1.	2.	3.	4.	5.
1. Disgust	942	3.74	0.81	0.8					
2. Shame	940	4.00	0.77	0.77	.78***				
3. Cleanliness norm	927	4.16	0.59	0.69	.51***	.51***			
4. Environmental belief	901	2.82	0.84	0.7	.02	.03	.03		
5. Mean number of wears (pants)	912	3.49	1.00	0.84	-.15***	-.14***	-.08*	-.05	
6. Often wash few items	941	1.31	0.62	NA	.09**	.09**	.05	.02	-.07*

Note: n = number of observations; SD = standard deviation; α = Cronbach’s alpha; NA = not applicable

*p < .05

**p < .01

***p < .001.

To get an initial understanding of the data, a comparison of the average wash frequency within each group was performed. This was done by dividing each index category into quartiles and calculating the average wash frequency within each of these new four groups. A t-test was then used to test if the average wash frequency (per person) of the 25% of the lowest scoring participants (i.e. Q1) differed from the 25% highest scoring participants of each index (i.e. Q4). Since the amount of laundry might differ between adults and children laundering frequencies per person were also calculated for single household without children, see Table 2.

**Table 2:** Average number of wash cycles per month (per person) grouped according to lowest (Q1) and highest (Q4) index quartiles and the results from an independent t-test between the two groups.

	All data			Single households, no children		
Index category	Mean (Q1)	Mean (Q4)	t	Mean (Q1)	Mean (Q4)	t
Disgust	4.23	5.38	-4.05***	4.94	7.65	-3.6***
Shame	4.40	5.37	-3.55***	5.02	7.04	-3.19**
Cleanliness norm	4.53	5.40	-2.82**	5.79	7.35	-1.83
Environmental belief	4.82	4.98	-0.51	5.56	6.27	-0.96
Mean number of wears (pants)	6.03	4.52	4.41***	8.51	5.41	3.61***
Often wash few items	4.71	6.18	-5.14***	5.86	8.13	-3.2**

Note:

*p < .05

**p < .01

***p < .001.

As may be seen in Table 2, the number of wash programs performed per person each month differed between the two groups for all index categories except for environmental belief. The index variables and background variables were then introduced hierarchically in a linear regression model, in order of hypothesized theoretical relevancy, see Table 3. Note that the values for “Environmental belief” and “Mean number of wears (pants)” were reversed before they were introduced (i.e. a score of 5 was re-coded as 1, and vice versa). This was done since these two indexes were assumed to be negatively correlated with the number of washes per month.

**Table 3:** Zero-order correlation between predictor variables and wash frequency; Hierarchical introduction of psychological (Model 1), behavioural (Model 2), and background (Model 3) variables into a linear regression with wash frequency as dependent variable.

	Zero-order correlation	Model 1 (R^2^ = 0.017**)		Model 2 (ΔR^2^ = 0.027***)		Model 3 (ΔR^2^ = 0.315***)			
**Variables**	**Pearson’s r**	**β**	**p**	**β**	**p**	**B [95% CI]**	**β**	**p**	**VIF**
Disgust	.13***	0.14	0.013	0.12	0.03	0.92 [-0.03, 1.88]	0.09	0.059	2.79
Shame	.11***	-0.06	0.258	-0.08	0.162	-0.40 [-1.41, 0.61]	-0.04	0.438	2.77
Cleanliness norm	.09**	0.06	0.167	0.05	0.179	0.23 [-0.70, 1.15]	0.02	0.632	1.47
Environmental belief	.03	-0.02	0.47	-0.02	0.623	-0.14 [-0.68, 0.40]	-0.01	0.622	1.01
Mean number of wears (pants)	-.18***			0.13	<0.001	0.94 [0.48, 1.41]	0.12	<0.001	1.07
Often wash few items	.23***			0.09	0.005	1.39 [0.66, 2.12]	0.11	<0.001	1.01
Adults	-.21***					2.49 [1.70, 3.28]	0.20	<0.001	1.24
Children	-.12***					3.81 [3.24, 4.38]	0.42	<0.001	1.25
Age	-.06					-0.01 [-0.04, 0.02]	-0.01	0.625	1.12
Household income	-.03					0.36 [0.16, 0.56]	0.12	<0.001	1.42

Note: *p < .05

**p < .01

***p < .001; The significancy of ΔR^2^is based on a one-way ANOVA test between each subsequent model. Adj. R^2^ for Model 3 = 0.350.

Looking at Table 3, it is interesting to note that only a few of the predictors were statistically significant in Model 2. It is also interesting to notice that environmental belief was non-significant in all of the models. Additionally, two of the significant predictors in Model 3 were habitual traits, suggesting that previous instances of behaviour are a better predictor of future behaviours (rather than the psychological constructs). This suggests that these aspects would be worthwhile exploring in greater detail. In Study 2 we therefore aimed to explore and pinpoint *which* mediating behaviours/habits that could be used to predict wash frequency.

## Study 2

### Methods and participants

Based on the results from Study 1 a structured qualitative study was conducted. Here many of the questions from the survey were discussed in more detail, highlighting contextual factors influencing decisions on laundering practices. The interviews were also an opportunity for us to capture a wider array of specific behaviours preceding the need to run a wash program. Participants were once again recruited in collaboration with NOVUS, screened on their self-reported wash responsibility in their respective households, and selected based on the relevant background variables (e.g. socio-economic factors, living situation, age, etc.). The data collection started at the 13^th^ of March 2023 and was completed the 30^th^ of March 2023. Since the invited participants were part of the NOVUS-recruited national panel where informed consent is mandatory for participation. A written ethical approval for the work was sought from the Central Ethical Review Board in Gothenburg, who determined that the research was exempt from the requirements stated in the Swedish Ethical Review Act. Since the aim of this qualitative study was to capture examples of mediating behaviours that lead to high laundry turn-over, including any expressed rationales, participants were selected on a number of additional criteria: participants who expressed a higher level of discomfort towards wearing dirty clothing, who stated that that they often run the washing machine with only a few clothing items, or acknowledge that they washed their clothes after only using them a few times. Since the results from Study 1 indicated that the number of children within each household predicted wash frequency, participants were stratified into four different groups:

1. Participants living alone (no children)
2. Participants living together with another adult (no children)
3. Participants living together with another adult and one child (the child must be younger than 2 years old)
4. Participants living together with another adult and at least two children.

In total, 47 adults were invited to participate. Out of these, 39 participants chose to attend the interviews (with 8–11 participants in each group). Each group was then interviewed during a separate two-hour long group chat (no webcam). This ensured anonymity as well as minimized social desirability bias among the participants. The resulting chat was then transcribed, cleaned, and analysed using the NVIVO software (version 12, release 1.6.1.).

### Results and discussion

In general, the interviews provided contextual information for how the more interesting questions in the survey were interpreted (e.g. *“How do you know when it is time to wash*?*”*, and *“When is clothing considered dirty*?*”*), as well as explicit reasons for specific laundry decisions. Four new categories of behaviours that could serve as potential mediators were identified:

- **Evaluation criteria:** To what extent a clothing item with stains and/or odour was deemed dirty.
- **Inadequate laundry loads:** To what extent the machine was filled up completely before running a program (i.e. as a complement to how often a machine was run with one or few garments).
- **Number of wears:** How many times specific clothing items could be used before they were thrown in the laundry basket (i.e. more types of apparel than just pants).
- **Bed linen change frequency:** How many nights bed linen could be used before it was thrown in the laundry basket.

These results were then used to re-frame some of the initial survey items and expand the number of questions regarding additional behaviours that might precede laundering.

## Study 3

### Methods and participants

Following the insights from Study 1 and 2, a revised and updated questionnaire was distributed to a new set of participants recruited from the NOVUS national panel. The data collection started at the 12^th^ of June 2023 and was completed the 22^nd^ of June 2023. As for Study 1 and 2, informed consent was ensured by NOVUS, and the Central Ethical Review Board in Gothenburg determined that the research was exempt from the requirements stated in the Swedish Ethical Review Act. In total 1136 people took part, out of which responses from 927 participants were used in the analysis after applying the same screening criteria as for Study 1. Compared to the initial survey, the updated version differed both in focus as well as direct formulations of the questions: the disgust index was extended to include an applicable item from the more general disgust scale [50,51] and one item used in the Body Odor Disgust Scale [52], the exact wording for items concerning shame and norms were altered to better reflect how some consumers related to these topics, more nuanced questions regarding environmental identity [36] were included, and additional items were added to better capture specific habitual behaviours preceding the intention to run a machine (e.g. number of uses before washing specific clothes, or evaluation criteria for dirtiness). The complete list of specific questions used for the survey can be found in the supporting information. To minimize the risk of misinterpretation of the updated questions, a convenience sample of respondents not part of the panel was asked to test the new version of the survey before distribution (results not included in this analysis).

### Results and discussion

In the same way as for Study 1, reliability calculations were performed on each potential index variable. Some of the individual items did not seem to fit the overall index and were therefore excluded from the continuing analysis. After dropping these items the final reliabilities of each index were deemed satisfactory, see Table 4. Since the index for ‘Mean number of nights (bed linen)’ only consisted of a single item it was not relevant for consistency validation.

**Table 4:** Descriptive summaries and bivariate correlation matrix for each index.

Index category	n	Mean	SD	α	1.	2.	3.	4.	5.	6.	7.
1. Disgust	925	3.99	0.77	0.74							
2. Shame	925	3.89	0.84	0.77	.59***						
3. Cleanliness norm	915	4.13	0.66	0.65	.44***	.44***					
4. Environmental identity	926	3.18	0.97	0.85	-.04	-.04	.06				
5. Evaluation sensitivity	925	2.93	1.04	0.75	.35***	.33***	.20***	-.25***			
6. Mean number of wears (clothes)	926	3.00	0.65	0.61	-.30***	-.28***	-.19***	.12***	-.33***		
7. Inadequate laundry loads	927	3.81	0.66	0.63	-.14***	-.10**	.03	.16***	-.18***	.08*	
8. Mean number of nights (bed linen)	922	3.12	0.73	NA	-.19***	-.15***	-.10**	.01	-.09**	.47***	.06

Note: n = number of observations; SD = standard deviation; α = Cronbach’s alpha; NA = not applicable

*p < .05

**p < .01

***p < .001.

Table 5 illustrates the results from the t-test within each index. Once again, the number of wash programs performed per person each month differed between the two quartiles for all index categories, except for environmental identity. These findings are consistent with Study 1, and it is interesting to note that pattern for environmental aspects remains even though the focal area has changed from environmental beliefs to environmental identity.

**Table 5:** Average number of wash cycles per month (per person) grouped according to lowest (Q1) and highest (Q4) index quartiles, and the result from an independent t-test between the two groups.

	All data			Single household, no children		
Index category	Mean (Q1)	Mean (Q4)	t	Mean (Q1)	Mean (Q4)	t
Disgust	4.33	5.61	-4.48***	4.93	6.74	-2.55*
Shame	4.50	5.60	-3.4***	5.56	7.25	-2.16*
Cleanliness norm	4.77	5.50	-2.08*	5.35	7.35	-2.65**
Environmental identity	5.19	4.75	1.23	6.26	5.83	0.49
Evaluation sensitivity	4.08	5.70	-4.96***	4.98	7.21	-2.79**
Mean number of wears (clothes)	6.37	4.20	5.75***	8.46	4.89	3.81***
Inadequate laundry loads	6.70	4.11	6.48***	7.94	5.06	3.25**
Mean number of nights (bed linen)	6.60	4.84	4.59***	9.40	5.90	3.64***

Note: *p < .05

**p < .01

***p < .001.

Table 6 presents the correlations between the self-reported number of monthly washes of laundry in the household (per person) and each updated index variable, including background variables. The table also depicts the results from hierarchically introducing each variable into a linear regression model with the average household wash frequency as dependent variable. The values for “Environmental identity”, “Mean number of wears (clothes)”, “Inadequate laundry loads”, and “Mean number of nights (bed linen)” were reversed before introduced (i.e. a score of 5 was recoded as 1, and vice versa). This was done since these indexes were assumed to be negatively correlated with the number of washes per month.

**Table 6:** Zero-order correlation between predictor variables and wash frequency; Hierarchical introduction of psychological (Model 1), behavioural (Model 2), and background (Model 3) variables into a linear regression with wash frequency as dependent variable.

	Zero-order correlation	Model 1 (R^2^ = 0.015**)		Model 2 (ΔR^2^ = 0.033***)		Model 3 (ΔR^2^ = 0.240***)			
**Variables**	**Pearson’s r**	**β**	**p**	**β**	**p**	**B [95% CI]**	**β**	**p**	**VIF**
Disgust	.14***	0.06	0.129	<0.01	0.915	0.25 [-0.51, 1.01]	0.02	0.516	1.70
Shame	.11***	0.01	0.774	-0.03	0.55	-0.15 [-0.86, 0.55]	-0.02	0.669	1.69
Cleanliness norm	.06	0.04	0.315	0.03	0.381	0.11 [-0.67, 0.90]	0.01	0.778	1.35
Environmental identity	-.05	0.07	0.027	0.03	0.371	0.03 [-0.45, 0.52]	<0.001	0.895	1.10
Evaluation sensitivity	.16***			0.13	<0.001	0.51 [0.01, 1.01]	0.07	0.047	1.38
Mean number of wears (clothes)	-.24***			0.08	0.053	1.17 [0.31, 2.03]	0.1	0.008	1.53
Inadequate laundry loads	-.26***			0.07	0.048	2.05 [1.33, 2.77]	0.17	<0.001	1.11
Mean number of nights (bed linen)	-.17***			0.03	0.366	0.55 [-0.16, 1.25]	0.05	0.127	1.32
Adults	-.23***					2.61 [1.87, 3.34]	0.22	<0.001	1.22
Children	-.15***					3.09 [2.50, 3.68]	0.34	<0.001	1.28
Age	.01					-0.02 [-0.05, 0.01]	-0.05	0.116	1.16
Household income	-.05					0.28 [0.09, 0.47]	0.09	0.005	1.29

Note: The significancy of ΔR^2^ is based on a one-way ANOVA test between each subsequent model.

*p < .05

**p < .01

***p< .001. Adj. R^2^ for Model 3 = 0.277.

Similar to the results from Study 1, none of the four psychological indexes turned out to be significant when accounting for background variables, see Table 6. The results from the interviews in Study 2 indicated however that each index might not predict washing per se but might rather be mediated through preceding behaviours. For example, few people would say that they choose to *run* the washing machine due to environmental reasons alone. Instead, the choice to wash is more likely to be prompted by specific triggers such as the need for a specific item, or a full laundry basket. This perceived need, or the choice to throw clothes into the basket to begin with (i.e. evaluation criteria), is however likely to be influenced by psychological constructs tied to disgust, shame, cleanliness norms, and environmental identity.

To test the possibility of mediation, the data was analysed using the PROCESS-macro [53]: psychological indexes were selected as independent variables (IV), behavioural indexes as mediators (MED), and wash frequency as dependent variable (DV). The results suggested a number of significant pathways between the independent variables and mediators, including significant indirect effect between the IV and DV, see Table 7.

**Table 7:** Mediation paths when treating the psychological aspects as independent variables (IV), stated behaviours as mediators (MED), and monthly wash frequency as dependent variable (DV).

	IV: Disgust		IV: Shame		IV: Clean. norm		IV: Env. identity	
Mediator (MED)	IV ⟶ MED	MED ⟶ DV	IV ⟶ MED	MED ⟶ DV	IV ⟶ MED	MED ⟶ DV	IV ⟶ MED	MED ⟶ DV
Evaluation sensitivity	0.352***	0.067*	0.35***	0.073*	0.178***	0.071*	0.243***	0.074*
Number of wears (clothes)	0.294***	0.097**	0.272***	0.101**	0.193***	0.098**	0.137***	0.101**
Inadequate laundry loads	0.131***	0.169***	0.091**	0.17***	-0.029	0.174***	0.157***	0.17***
Number of nights (bed linen)	0.174***	0.049	0.124***	0.05	0.096**	0.053	0.02	0.05

Note: *p < .05

**p < .01

***p < .001.; The bootstrapped total standardized indirect effects of: Disgust: 0.083 [0.052, 0.117], Shame: 0.075 [0.047, 0.105], Cleanliness norm: 0.031 [0.009, 0.056], and Environmental identity: 0.06 [0.036, 0.087].

The results illustrated in Table 7 support the idea that washing frequency is influenced by our suggested psychological constructs, mediated through a set of preceding behaviours. However, while these results are interesting some methodological challenges remain that need to be addressed. The validity of each index relies on Cronbach’s alpha as a statistical criterion for internal consistency. While this is common practice, it would be wrong to assume that correlation between a set of variables means that they also originate from a common factor [54]. In addition, the mediation calculations performed by the PROCESS-macro for Table 7 test each IV separately. This means that potential overlaps between each IV are not accounted for. Since highly correlated IVs have the potential to cancel each other out, even if each IV previously has been shown to exert a direct and/or indirect effect on the DV [53], the very high bivariate correlation between the construct of shame with disgust and cleanliness norms was of special concern, see Tables 1 and 4.

**Fig 1:**
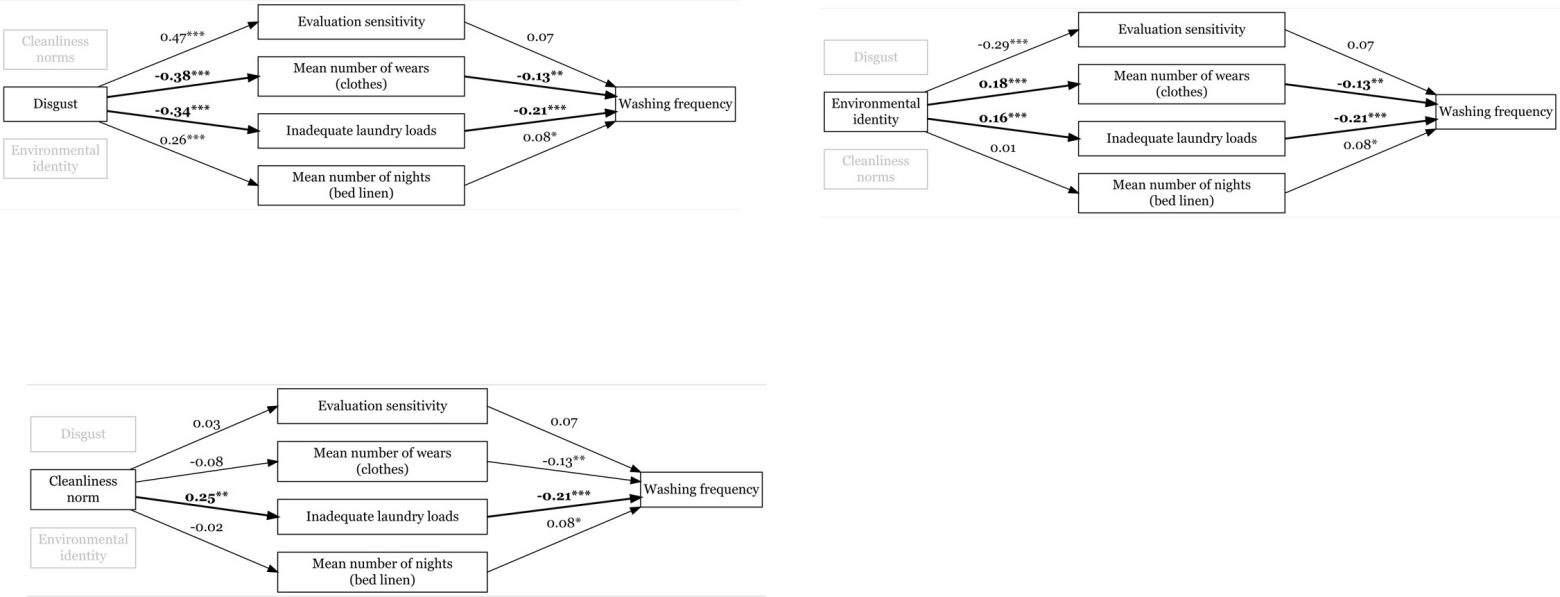
Resulting a-paths (IV->MED) and b-paths (MED->DV) when using a SEM-model that include all three IVs at the same time. (A) The mediation of disgust. (B) The mediation of cleanliness norms. (C) The mediation of environmental identity. *p < .05, **p < .01, ***p < .001. Significant bootstrapped standardized indirect paths in bold.

To account for these challenges a more robust structural equation model (SEM) was tested using the lavaan library in R. Instead of creating index values based on the arithmetic means, each psychological construct and mediating behaviour was treated as a latent variable represented by the measured items. As expected, the result highlighted a number of challenges including 6 bootstrap errors (out of 1000) for the complete latent construct model (i.e. when including all of the 4 IVs, 4 MEDs, and 1 DV while controlling for the background variables). The correlations between shame and disgust (*r* = 0.799 and between shame and cleanliness norms (*r* = 0.676) were still high in the latent SEM-model, indicating a possible multicollinearity and/or suppression effects between the Ivs. To test whether the SEM-model may be over-specified, we ran an updated model where the latent construct of shame was removed. This revised model yielded no bootstrap errors and revealed a number of significant paths between the latent constructs and the DV, see Fig 1A–1C. The measures of chi squared divided by the degrees of freedom (*X*^2^/*d*.*f*. = 3.852), root mean square error of approximation (RMSEA = 0.055), and standardized root mean square residual (SRMR = 0.056) indicated an acceptable model fit [55,56]. However, the comparative fit index (CFI = 0.865) just missed the threshold. Although the model fit could be increased by adding undefined latent constructs, we decided against such a bottom-up approach since the point of departure of this research is theory based. Five indirect paths between the IVs and DV turned out to be significant, see Table 8.

**Table 8:** Significant standardized bootstrapped estimates for indirect effects on wash frequency using a latent construct SEM-model. Confidence interval in brackets.

IV	MED	Std. indirect effect on DV
Disgust	Mean number of wears (clothes)	0.051 [0.009, 0.093]
Disgust	Inadequate laundry loads	0.071 [0.024, 0.118]
Norm	Inadequate laundry loads	-0.053 [-0.094, -0.011]
Environmental identity	Mean number of wears (clothes)	-0.025 [-0.045, -0.004]
Environmental identity	Inadequate laundry loads	-0.033 [-0.057, -0.01]

Since the SEM model includes previously unverified items, the resulting estimates of each indirect effect need to be interpreted with some caution. Still, the general direction for each standardized indirect effect between the IVs and DV is clear. Increased levels of disgust sensitivity suggest a higher wash frequency, whereas increased levels of environmental identity would reduce the number of washes per month, see Table 8. As for cleanliness norms, the SEM-model suggested that an increased sensitivity leads to a decreased wash frequency (which is contrary to the results from the zero-order correlation and PROCESS-model). Possible explanations of this could be additional suppression effects from the latent construct of disgust, reversed causality (i.e. people wash more frequently to reduce their fear of cleanliness norm violations), or simply that the specific items used for the construct were insufficient.

Regardless, the consistent findings in the PROCESS-model and the SEM-model are significant mediated effects of disgust sensitivity and environmental identity on wash frequency. Moreover, these effects are at odds with each other, meaning there is a high probability of conflicting goals when evaluating whether a specific clothing item should be put in the laundry basket or reused.

## General discussion

The results presented in this paper illustrate that excessive laundering is influenced by psychological constructs mediated through a set of behaviours. Looking at each construct separately, increased sensitivity to disgust, shame, or cleanliness norm violations were constructs associated with a higher washing frequency per person (see Tables 2 and 5). Interestingly, no such effects could be observed for environmental belief or environmental identity. The same dynamic was revealed when reviewing the zero-order correlations between the constructs and the general washing frequency (see Tables 3 and 6). However, when introduced into a linear regression accounting for behavioural and background variables all these effects seemed to disappear. Initially this was thought to be a result of the expected low discriminatory validity commonly associated with introspective measurements. Later investigations using the PROCESS-macro instead showed that the effects did not, in fact, disappear but were rather mediated through a set of preceding behaviours, see Table 7. These mediating behaviours (such as washing few items, labelled “Inadequate laundry loads” in the analyses) were exercised before the actual act of running the washing machine. Some of these mediated paths persisted even after controlling for potential overlaps between each IV in a latent construct model using SEM, see Table 8.

One interesting finding is the relatively large influence of disgust (compared to the other psychological constructs). This is intuitively valid, e.g. we can all remember occasions of washing disgusting clothes. But what is often overlooked is that disgust suggests a potential influence from the behavioural immune system (BIS) regarding laundering behaviours. The BIS is considered an initial behavioural defence against harmful parasites and pathogens [57]. Through a number of psychological mechanisms, the BIS detects cues for the presence of infectious pathogens, triggers the relevant emotional, cognitive, and/or behavioural response, and by extension averts a potential infection [58]. However, since pathogens are invisible to the human eye the focus of the system becomes to identify the *potential* presence of infectious agents through superficial visual, auditory and olfactory cues [59]. Since mistakes in proper aversive responses can be costly from an evolutionary perspective, it is better to fail on the side of caution. This means that individuals by default are more prone to evaluate an object (or a person) as contaminated even when that is not the case [17]. Empirical evidence implies that the BIS has implications for person perception, intergroup prejudice, mate preferences, sexual behaviour and conformity [60]. With regard to domestic laundering, a common example for triggering a BIS response is failing to adhere to prevailing norms, especially concerning customary hygiene practices [58,60].

Our results suggest that higher levels of disgust sensitivity may lead to higher washing frequencies, whereas higher levels of pro-environmental identity may lead to lower washing frequencies, see Table 8. Given this, it is not surprising that previous interventions to steer laundering decisions have failed. In many cases the rationale for changed behaviour has been motivated solely from a pro-environmental perspective. Although well intended, we propose that such arguments are only valid for people who experience a net positive effect from feeling pro-environmental at the cost of feeling disgust. Furthermore, we argue that the assumed effects of the BIS amplify excessive laundering. Failure to adhere to what is considered “a minimum level” of cleanliness practices would increase the risk that you inadvertently signal an higher level of pathogenic presence, potentially resulting in social stigmatization [61]. If this dynamic is intuitively understood by people, the amount of laundering would gradually increase since it is better to err at the side of caution. This would also mean that it would be nearly impossible to motivate a deviation from current practices through pro-environmental arguments alone; especially if the alternative practice offers few advantages other than perhaps a cleaner conscience. Many consumers seem to believe that washing in a more environmentally sound way reduces the machines capacity to remove stains and/or odours.

For successful public policies targeting emissions from laundering, several aspects need to be considered. First, it might seem trivial but for pro-environmental messages to be effective in changing behaviour, the underlying motivation for that specific behaviour must be rooted in environmental concerns. Unfortunately this is not the case for domestic laundering. No-one runs the washing machine for environmental reasons alone. Instead, most people wash their clothes for more practical reasons: they need a specific item, the laundry basket is full, or it is the only time during the week that washing is possible. One way to solve this could instead be to target the preceding decisions that slowly generate the need to wash, treating reduced emissions as a co-benefit rather than the main objective. Some examples of such behaviours are listed in this article, e.g. evaluation criteria for when clothes are considered dirty (i.e. how quickly the laundry basket is filled up) or whether only few items are loaded into the washing machine.

Looking ahead, some important challenges remain that need to be addressed. The psychological constructs need further validation both regarding the specific connotations used and for potential cultural variations. For example, individuals from societies that pride honour-related values are more prone to feel shame, whereas people from societies that treasure individualistic values are more inclined to feel guilt [62]. Due to this, it is likely that many of the respondents answering our survey questions regarding shame instead would feel guilt, since Swedish values are traditionally more individualistic [63]. Feelings of shame are linked with disgust, but guilt is instead linked with social cues of anger [64]. This would suggest that laundering behaviour in Sweden could be more effectively influenced by social cues connected to guilt and anger, rather than disgust and shame. However, whether this is the case remain to be investigated. Likewise, it is unclear if the results could be replicated in other non-western societies (e.g. in Africa or Asia).

## Conclusions

Policies trying to enhance pro-environmental behaviour will inevitably force consumers to prioritize competing interests. In this article we argue that people are confronted with an implicit dilemma when deciding whether to wash or not: reducing emissions but risking social repercussions. Since the latter take priority for the general consumer, it comes as no surprise that previous interventions have been unsuccessful in steering behaviour. Of special interest is the potential influence of the emotion of disgust for cleanliness evaluations regarding clothes, and by extension wash frequency. Specific policy recommendations for laundering include treating reduced emissions as a beneficial by-product rather than the main objective. This means focusing more on the underlying behaviours that create a need to wash rather than the act of running the washing machine. More specifically, efforts should be made to extend the use frequency of clothes between washes by desensitizing feelings of disgust. Further avenues to be explored include a better understanding of the relative importance of shame and guilt for laundering behaviours. This would allow for better adjustments of pro-environmental initiatives when these are deployed in either honour-related societies, or societies that emphasize individualistic values.

## Supporting information

S1 Appendix Survey questions used in Study 1. (DOCX)

S2 Appendix Survey questions used in Study 3. (DOCX)
