# Supplementary material for: Pro-environmental behaviour is undermined by disgust sensitivity: The case of excessive laundering
Source: PLoS One. 2024 Jun 13;19(6):e0302625. doi: 10.1371/journal.pone.0302625 (PMC11175451; doi:10.1371/journal.pone.0302625)
Supplement: S1 Appendix — (DOCX) [file pone.0302625.s001.docx]

*NOTE The following table list a translation of the most relevant questions collected in Survey 2. Additional information can be found in the appended datasets, including the complete list of questions used in the survey.*

| **Index** | **Data file ID** | **Question (translation from Swedish)** |
| --- | --- | --- |
|  |  | **To what extent do you agree to the following statements?** *Likert scale (Strongly disagree – Strongly agree)* |
| Disgust 1 | G11_SambandsmodellN1 | I feel uncomfortable when people near me wear clothes with stains. |
| Disgust 2 | G11_SambandsmodellN2 | I feel uncomfortable when people near me wear clothes that smell bad. |
| Disgust 3 | G11_SambandsmodellN3 | The thought of wearing clothes with stains in everyday settings is something that triggers discomfort/uneasiness |
| Disgust 4 | G11_SambandsmodellN4 | I do not like wearing clothes that look dirty, even if they are newly washed |
| Disgust 5 | G11_SambandsmodellN5 | The thought of wearing clothes that smell, in an everyday setting, is something that triggers discomfort/uneasiness |
| Shame 1 | G11_SambandsmodellN17 | I would feel ashamed if I discovered that my clothes had stains |
| Shame 2 | G11_SambandsmodellN18 | I would feel ashamed if I discovered that my clothes smelled bad |
| Shame 3 | G11_SambandsmodellN19 | I think people would have disapproved if I had worn clothes with stains |
| Shame 4 | G11_SambandsmodellN20 | I think people would have disapproved if I had worn clothes that smelled bad |
| Cleanliness norm 1 | G11_SambandsmodellN13 | Most people I know think it is important to avoid wearing clothes with stains. |
| Cleanliness norm 2 | G11_SambandsmodellN14 | Most people wear very clean clothes in everyday settings |
| Cleanliness norm 3 | G11_SambandsmodellN15 | My friends and colleagues wear very clean clothes |
| Cleanliness norm 4 | G11_SambandsmodellN16 | Most people I know think it is important to avoid wearing clothes that smell bad. |
| Environmental belief 1 | G11_SambandsmodellN8 | Washing clothes damages the environment. |
| Environmental belief 2 | G11_SambandsmodellN9 | My way of washing clothes damages the environment. |
| Behavioural robustness 1 | G11_SambandsmodellN10 | The way I do the laundry differs little from week to week |
| Behavioural robustness 2 | G11_SambandsmodellN11 | I do not need to actively think when I do the laundry |
| Behavioural robustness 3 | G11_SambandsmodellN12 | My actions are automatic when I do the laundry. |
| Often wash few items | G10_EkonomiN2 | I often run a washing machine with only one or a few items in it. |
|  |  |  |
|  |  | **Before you wash your pants, how many times have you worn your…**  *Scale (Once – 11 times or more)* |
| Mean number of wears (pants) 1 | S15_Anvandning_typN1 | *… jeans?* |
| Mean number of wears (pants) 2 | S15_Anvandning_typN2 | *… leggings/tights?* |
| Mean number of wears (pants) 3 | S15_Anvandning_typN3 | *… chinos?* |
| Mean number of wears (pants) 4 | S15_Anvandning_typN4 | *… suit pants?* |
