# Supplementary material for: Pro-environmental behaviour is undermined by disgust sensitivity: The case of excessive laundering
Source: PLoS One. 2024 Jun 13;19(6):e0302625. doi: 10.1371/journal.pone.0302625 (PMC11175451; doi:10.1371/journal.pone.0302625)
Supplement: S2 Appendix — (DOCX) [file pone.0302625.s002.docx]

*NOTE The following table list a translation of the most relevant questions collected in Survey 2. Additional information can be found in the appended datasets, including the complete list of questions used in the survey.*

| **Index item** | **Data file ID** | **Question (translated from Swedish)** |
| --- | --- | --- |
|  |  | **To what extent do you agree to the following statements?** *Likert scale (Strongly disagree – Strongly agree)* |
| Disgust 1 | Q6_7N1 | I think it is annoying when people near me wear clothes that smell of tobacco or food. |
| Disgust 2 | Q6_7N2 | The thought of having to wear clothes that I know are stained when I go to work/school is distressing. |
| Disgust 3 | Q6_7N3 | The thought of having to wear clothes that have a strange smell when I go to work/school is distressing. |
| Disgust 4 | Q6_7N4 | It is okay to fish a garment out of the washing basket just before going to work/school and put it on (without washing or airing it) |
| Disgust 5 | Q6_7N5 | It is OK to put clothes into the wardrobe even if you have used them more than 4 times, assuming the clothes do not contain stains or unusual odour |
|  |  |  |
|  |  | **How disgusting you would find the following experiences?** *Likert scale (Not disgusting at all – Very disgusting)* |
| Disgust 6 | Q8N1 | You discover that a friend of yours changes underwear only once a week. |
| Disgust 7 | Q8N2 | You are standing next to a stranger and notice that the T-shirt they are wearing smells from their sweat |
|  |  |  |
|  |  | **To what extent do you agree to the following statements?** *Likert scale (Strongly disagree – Strongly agree)* |
| Shame 1 | Q6_7N10 | I would feel ashamed if someone at work/school told me that my clothes had a subtle smell of sweat |
| Shame 2 | Q6_7N11 | I would feel ashamed if someone at work/school told me that my clothes had a subtle smell of tobacco or food |
| Shame 3 | Q6_7N12 | I would feel ashamed if someone at work/school told me that my clothes had a visible stain |
| Shame 4 | Q6_7N13 | It is important that my children have clean clothes when we leave home, for example going to the store, kindergarten, or meeting friends |
| Shame 5 | Q6_7N14 | It is important for me to feel that the clothes I wear outside my home are clean |
| Cleanliness norm 1 | Q6_7N6 | The people I meet at work/school expect that I wear clean clothes |
| Cleanliness norm 2 | Q6_7N7 | Most people wear very clean clothes in everyday settings |
| Cleanliness norm 3 | Q6_7N8 | It is OK to use clothes that contain stains, as long as the stains are not visible |
| Cleanliness norm 4 | Q6_7N9 | Most people I know feel that it is important to wear clothes that feel fresh/newly washed, when meeting other adults outside home |
| Environmental identity 1 | Q6_7N15 | It is important to minimize environmental impacts when washing |
| Environmental identity 2 | Q6_7N16 | My way of washing clothes is hurtful for the environment |
| Environmental identity 3 | Q6_7N17 | I see myself as an environmentalist |
| Environmental identity 4 | Q6_7N18 | I am pleased to be an environmentalist |
| Environmental identity 5 | Q6_7N19 | I feel strong ties with environmentalism |
| Environmental identity 6 | Q6_7N20 | I identify with other environmentalists |
| Environmental identity 7 | Q6_7N21 | If the stains or odour remain after washing my clothes, I might as well throw away the specific clothing item and replace it with a new one |
| Evaluation sensitivity 1 | Q9N1 | If a garment has gotten a stain I throw it in the laundry basket, even if it would be possible to remove the stain by hand |
| Evaluation sensitivity 2 | Q9N2 | If a garment has gotten stained, I throw it in the laundry basket, even if the stain would be possible to hide |
| Evaluation sensitivity 3 | Q9N3 | If a garment has gotten an unusual odour I throw it in the laundry basket, even if it would be possible to eliminate the smell by airing the garment. |
| Evaluation sensitivity 4 | Q14N2 | Clothes that have become moist from sweat need to be cleaned the same day |
|  |  |  |
|  |  | **How many times have you worn…** *Scale (Once – 11 times or more)* |
| Mean number of wears (clothes) 1 | Q10N1 | …the pants that you use at work/school, before throwing them into the laundry bin? (regardless of if you use jeans, chinos, suite pants etc.) |
| Mean number of wears (clothes) 2 | Q10N2 | … the upper body clothing items closest to your body that you use at work/school, before throwing them into the laundry bin? (regardless of if you use t-shirts, shirts, blouses etc.) |
| Mean number of wears (clothes) 3 | Q10N3 | …the second layer clothing items that you use at work/school, before throwing them into the laundry bin? (regardless of if you use cardigans, sweatshirts, etc.) |
|  |  |  |
|  |  | **To what extent do you agree to the following statements?** *Likert scale (Strongly disagree – Strongly agree)* |
| Mean number of wears (clothes) 4 | Q11N4 | I always dress myself with a new set of clothes after taking a shower (if I do not go to bed) |
| Inadequate laundry loads 1 | Q13 | Which of the pictures best describes how you usually load your laundry machine |
| Inadequate laundry loads 2 | Q14N1 | I/my household often run a wash program with one, or few, items |
| Inadequate laundry loads 3 | Q14N3 | I/my household usually wash full machines |
|  |  |  |
| Mean number of nights (bed linen) | Q12 | **I/we change the bed linen…**  *Scale (After a couple of days – After longer time than a month)* |
